# Case Report: Late-Onset Mitochondrial Disease Uncovered by Metformin Use in a Patient With Acute Verbal Auditory Agnosia

**DOI:** 10.3389/fneur.2022.863047

**Published:** 2022-03-25

**Authors:** Wei-Hao Lin, I-Hsiao Yang, Hui-En Cheng, Hsiu-Fen Lin

**Affiliations:** ^1^Department of Neurology, Kaohsiung Medical University Hospital, Kaohsiung, Taiwan; ^2^Department of Medical Imaging, Kaohsiung Medical University Hospital, Kaohsiung, Taiwan; ^3^Department of Neurology, College of Medicine, Kaohsiung Medical University, Kaohsiung, Taiwan

**Keywords:** MELAS, auditory agnosia, pure word deafness, metformin, case report

## Abstract

**Introduction:**

Verbal auditory agnosia is rarely caused by mitochondrial encephalopathy, lactic acidosis, and stroke-like episodes (MELAS) syndrome. Lactate acidosis, which is the adverse effect of metformin, has proposed links to mitochondrial dysfunction and may trigger clinical features of mitochondrial diseases.

**Case Presentation:**

A 43-year-old right-handed man presented to our emergency department with acute onset fever and headache accompanied by impaired hearing comprehension. He could communicate well through handwritten notes but could not understand what others were saying. He had been diagnosed as having diabetes mellitus 2 months prior to this event. Vildagliptin 100 mg/day and metformin 1,700 mg/day were prescribed for glucose control. Laboratory tests revealed elevated lactate levels in serum and cerebrospinal fluid of the patient. Brain MRI disclosed bilateral temporal lesions. Acute encephalitis with temporal involved was initially diagnosed and acyclovir was given empirically. However, follow-up MRI after acyclovir treatment revealed a progression of prior lesions. Further mitochondrial genome analysis revealed a mitochondrial DNA point mutation at position 3,243 (m.3243A > G) with 25% heteroplasmy, which is compatible with MELAS. His clinical symptoms and serum lactate levels were improved after discontinuing the metformin use.

**Conclusions:**

To our knowledge, this is the first report of a patient having late-onset MELAS syndrome that manifested as acute verbal auditory agnosia, which was identified after the patient began using metformin. Metformin is known to inhibit mitochondrial function and could trigger clinical features of MELAS syndrome. We encourage clinicians to maintain a high level of awareness that diabetes mellitus can be caused by mitochondrial disease and to exercise caution in the prescription of metformin.

## Introduction

Verbal auditory agnosia is an auditory recognition impairment specific to speech sounds. It is most commonly caused by cerebrovascular accidents. On the other hand, mitochondrial encephalopathy, lactic acidosis, and stroke-like episodes (MELAS) syndrome are rarely the cause (1). Metformin is widely used as a first-line antidiabetic drug. However, it is associated with the rare side effect of lactic acidosis, which has proposed links to mitochondrial dysfunction (2). Herein, we reported a rare case of acute verbal auditory agnosia, which was detected after metformin use, in an individual with late-onset MELAS syndrome.

## Case Presentation

A 43-year-old right-handed man presented to our emergency department with acute onset fever and headache accompanied by impaired hearing comprehension, auditory hallucination, and mild paraphasia. No focal weakness or meningeal irritation signs were noted. The functions of naming, speech fluency, writing, and reading were preserved. He could communicate well with his family through handwritten notes but could not understand what others were saying. Although the patient experienced auditory speech recognition problems, he could recognize and distinguish music and environmental noises. According to his medical history, he had been diagnosed as having diabetes mellitus 2 months prior to this event. Vildagliptin 100 mg/day and metformin 1,700 mg/day were prescribed for glucose control.

**Figure 1:**
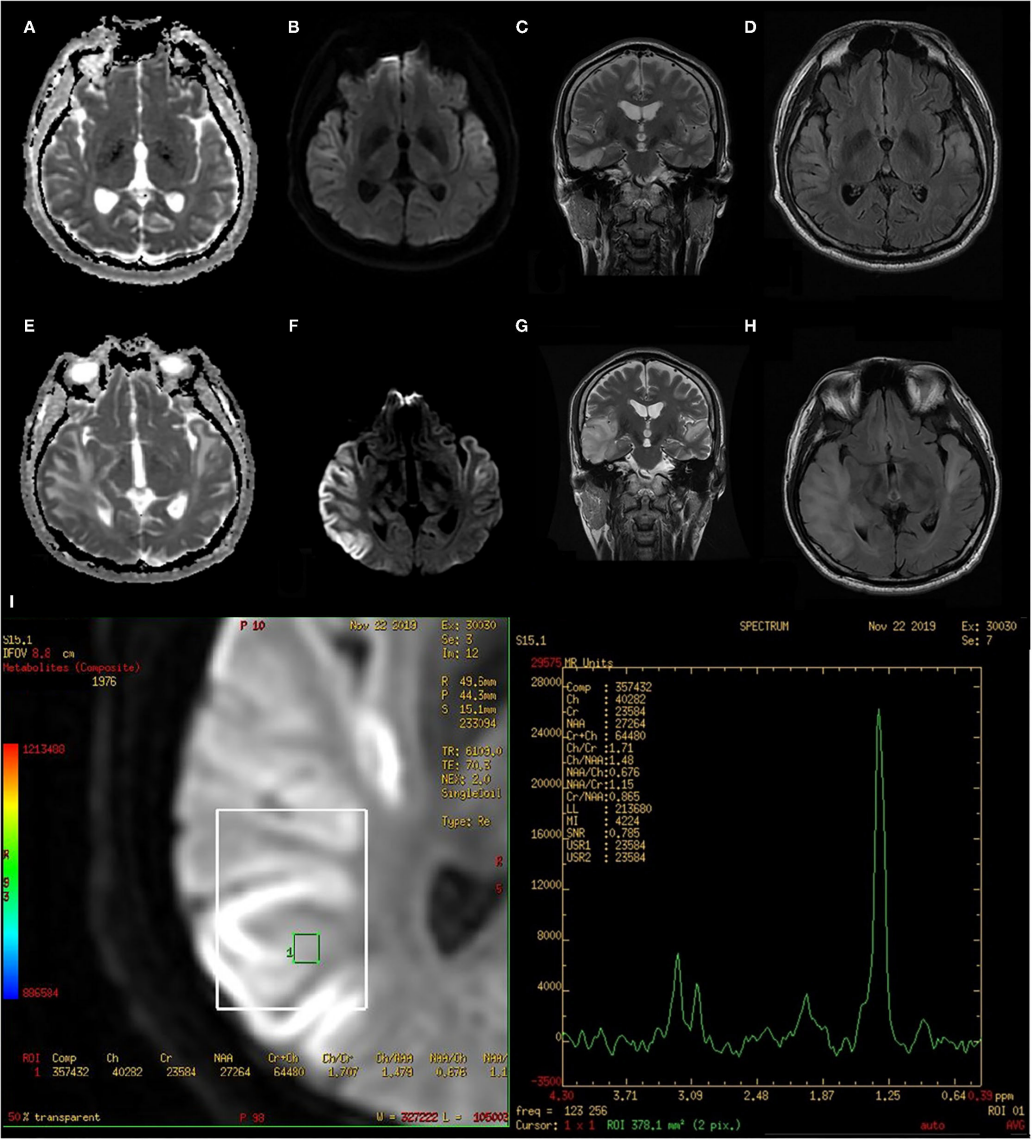
Initial and follow-up MRI after 14 days of acyclovir treatment. **(A)** initial ADC, **(B)** initial DWI, **(C)** initial T2 corona, **(D)** initial T2 FLAIR, **(E)** follow-up ADC, **(F)** follow-up DWI, **(G)** follow-up T2 corona, **(H)** follow-up T2 FLAIR, and **(I)** follow-up MRS. ADC, Apparent diffusion coefficient; DWI, Diffusion-weighted image; FLAIR, Fluid attenuated inversion recovery; MRS, Magnetic resonance spectroscopy.

Laboratory analysis revealed elevated creatine phosphokinase (CPK) (280 IU/L) and lactate (5.6 mmol/L) levels. The patient's leukocyte and C-reactive protein levels were within the normal range. Brain CT revealed a hypodense lesion at the right temporal lobe. Brain MRI revealed lesions at the bilateral superior temporal gyrus and right inferior temporal gyrus, with hyperintensity on a T2-weighted image, hypointensity on a T1-weighted image without gadolinium enhancement, and restricted diffusion on a diffusion-weighted image (Figures 1A–D). Pure-tone audiometry revealed mild hearing impairment bilaterally across all frequencies, which would not affect the performance of activities of daily living. Finally, the brainstem auditory evoked potential has shown a poor wave pattern.

Due to the presence of fever, headache, and verbal auditory agnosia, as well as bitemporal lobe involvement on brain images, acute viral encephalitis was suspected. Cerebrospinal fluid (CSF) studies revealed a cell count of 2 cell/μL, a glucose concentration of 88 mg/dL (serum 182 mg/dL), a protein concentration of 33 mg/dL, and elevated lactate (5.9 mmol/L). We empirically prescribed a regimen of intravenous acyclovir for 14 days. On the 14th day, CSF studies were followed by acyclovir treatment which revealed an increased lactate level (4.6 mmol/L). The patient's serum lactate level returned to being abnormally high (5.7 mmol/L). A follow-up MRI after the acyclovir treatment revealed a progression of prior lesions across the bilateral temporal lobes (Figures 1E–H).

During this hospitalization, the patient complained of chronic diarrhea since he started taking metformin during the previous 2 months. Because of this, we switched vildagliptin/metformin to linagliptin under the suspicion that metformin had an adverse effect. After metformin was ceased, a decrease in serum lactate (2.4 mmol/L) and CPK (135 IU/L) levels was noted in the follow-up data, and the patient's condition gradually improved.

Based on our clinical findings, mitochondrial etiology was considered. Magnetic resonance spectroscopy revealed an apparent lactate peak (Figure 1I). Direct sequencing analysis of the blood sample was performed by a polymerase chain reaction to screen the mitochondrial MT-TL1 gene and revealed a mitochondrial DNA point mutation at position 3243 (m.3243A > G) with 25% heteroplasmy, which is compatible with MELAS syndrome. No condition related to MELAS syndrome was noted in the patient's family history (Supplementary Figure 1). We discharged him, and with no further metformin use, the follow-up MRI 5 months later revealed a decreased lesion volume (Supplementary Figure 2), with communication function returning to baseline 7 months later.

## Discussion and Conclusion

To our knowledge, this is the first report of a patient having late-onset MELAS syndrome that manifested as acute verbal auditory agnosia, which was identified after the patient began using metformin.

Mitochondrial encephalopathy, lactic acidosis, and stroke-like episodes syndrome have diverse clinical manifestations, including myopathy, exercise intolerance, stroke-like episodes, seizure, elevated serum lactate levels, sensorineural hearing loss (SNHL), and diabetes mellitus (3). In more than 90% of cases, the onset occurs before age 40 years, and typically, it occurs before age 20 years (3, 4). Most auditory deficits detected in patients with MELAS are peripheral SNHL. Cortical auditory symptoms are seldom noted. Case reports have rarely described auditory agnosia as a presentation of stroke-like episodes in patients with MELAS syndrome (1, 5).

Auditory agnosia is a rare condition related to the impairment of auditory perception and recognition, and affected patients have relatively intact linguistic processing abilities, including reading, writing, and speaking. Patients are aware of sounds but have sound identification difficulties. The form of agnosia specific to speech perception is called verbal auditory agnosia. Typical lesions are usually located in the bilateral superior temporal cortical regions, but approximately 30% of cases are characterized by unilateral involvement and left lateralization (6). A cerebrovascular accident is the most common cause. When patients have temporal lobe lesions not confined to the vascular territory, different etiologies should be considered, including herpes simplex encephalitis and MELAS syndrome. Similar to our presented case, Smith et al. (1) had reported a patient with a stroke-like episode of acute auditory agnosia at age 61 with a low level of heteroplasmy in a genetic blood test. However, their patient had more typical clinical findings including chronic bilateral SNHL, underweight status, chronic migraine, and maternal family history. Miceli et al. (5) had also reported a young woman with MELAS who presented auditory agnosia without previous hearing deficits. Although SNHL was the mainly auditory symptom in patients with MELAS, cortical auditory disorders could be developed at any stage of the disease.

Our patient was relatively healthy prior to this acute event. We suspected MELAS syndrome due to the presence of persistently elevated lactate levels in the blood and CSF. In patients with MELAS syndrome, the inability of dysfunctional mitochondria to produce sufficient adenosine triphosphate results in inadequate glucose oxidization, thus causing pyruvate to accumulate and then shunt pyruvate to lactate. Lactate can either be oxidized by mitochondria or converted back to glucose through gluconeogenesis. Metformin is known to inhibit Complex I of the mitochondrial respiratory chain, and this may decrease lactate removal *in vivo* and inhibit mitochondrial oxidative phosphorylation *in vitro* (2). Because of its propensity to cause lactic acidosis, a recent review paper had suggested that metformin should be avoided in individuals with MELAS syndrome (7). On the contrary, a recent international consensus of experts considered that metformin was a safe medicine in mitochondrial disease (8). In the literature review, a couple of case reports had also raised the concern at the causal effect of metformin on stroke-like episodes in MELAS syndrome (9–11). Although the relationship between metformin and the stroke-like episode still remains speculative and uncertain, the metformin should be used cautiously as long as the acid-base status in blood needs to be regularly followed.

In conclusion, this patient with MELAS syndrome presented with rarely observed features of verbal auditory agnosia, which was initially misdiagnosed as herpes simplex encephalitis, and might be uncovered after the use of metformin. In the context of this patient's experience, we encourage clinicians to maintain a high level of awareness that diabetes mellitus can be caused by mitochondrial disease and to exercise caution in the prescription of metformin.

## Data Availability Statement

The original contributions presented in the study are included in the article/Supplementary Material, further inquiries can be directed to the corresponding author.

## Ethics Statement

Ethical review and approval was not required for the study on human participants in accordance with the local legislation and institutional requirements. The patients/participants provided their written informed consent to participate in this study. Written informed consent was obtained from the individual(s) for the publication of any potentially identifiable images or data included in this article.

## Author Contributions

W-HL treated the patient and drafted the manuscript. I-HY evaluated the brain image and revised the manuscript. H-EC treated the patient. H-FL treated the patient and revised the manuscript for intellectual content. All authors have read and approved the final submitted manuscript.

## Funding

The work was supported by Kaohsiung Medical University Hospital intramural grant to cover the fee for English proofreading.

## Conflict of Interest

The authors declare that the research was conducted in the absence of any commercial or financial relationships that could be construed as a potential conflict of interest.

## Publisher's Note

All claims expressed in this article are solely those of the authors and do not necessarily represent those of their affiliated organizations, or those of the publisher, the editors and the reviewers. Any product that may be evaluated in this article, or claim that may be made by its manufacturer, is not guaranteed or endorsed by the publisher.

## Abbreviations

MELAS: Mitochondrial encephalopathy, lactic acidosis, and stroke-like episodes
CPK: Creatine phosphokinase
MRI: Magnetic resonance imaging
CSF: Cerebrospinal fluid
SNHL: Sensorineural hearing loss.
