# Supplementary material for: Case Report: Late-Onset Mitochondrial Disease Uncovered by Metformin Use in a Patient With Acute Verbal Auditory Agnosia
Source: Front Neurol. 2022 Mar 25;13:863047. doi: 10.3389/fneur.2022.863047 (PMC8990297; doi:10.3389/fneur.2022.863047)
Supplement: Supplementary file 1 [file Presentation_1.pdf]

Supplementary data

Supplementary figure 1. Family pedigree

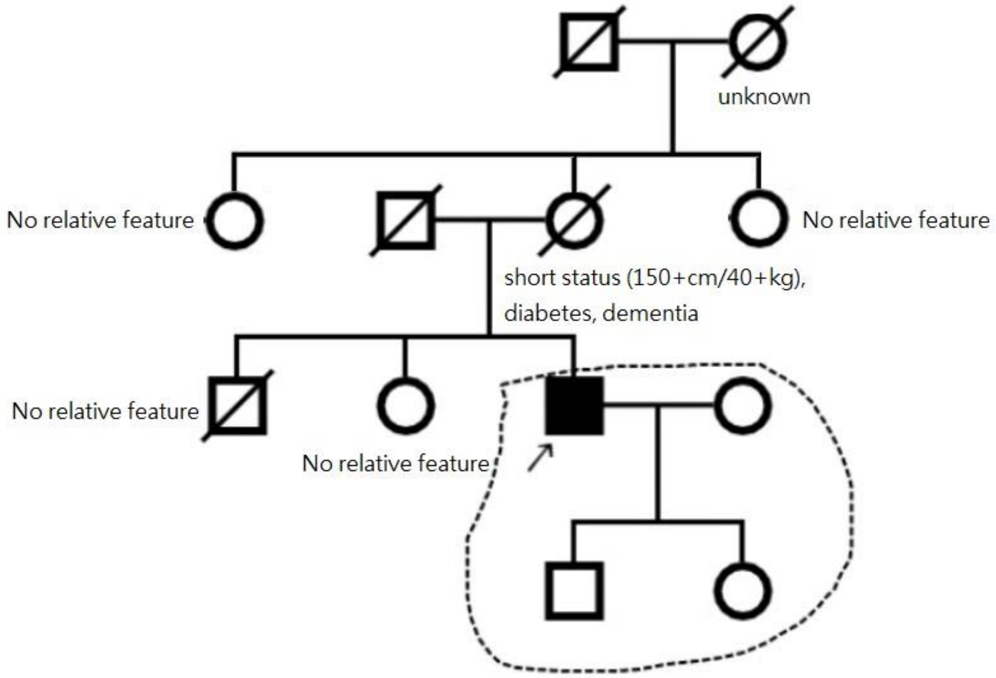

Supplementary figure 2. Follow-up brain MRI 5 months later

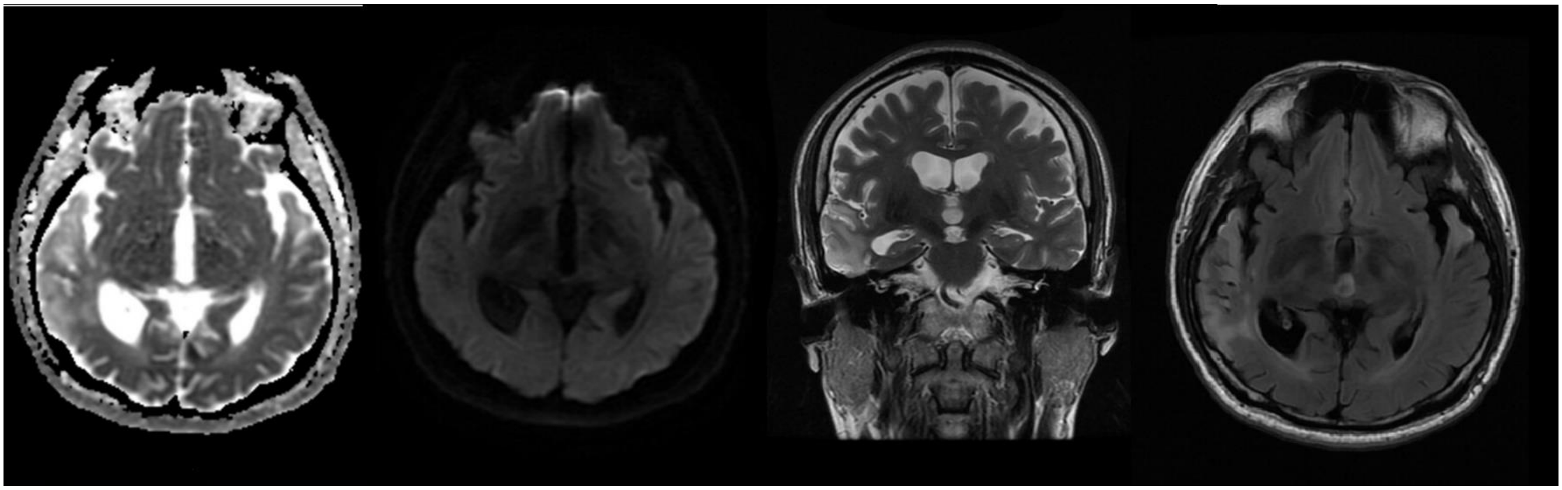

The images were arranged in ADC, DWI, T2WI, and T2 FLAIR.
